# Environmental sustainability in the online media discourses of Saudi Arabia: A corpus-based study of keyness, intertextuality, and interdiscursivity

**DOI:** 10.1371/journal.pone.0277253

**Published:** 2022-11-17

**Authors:** Shrouq Almaghlouth

**Affiliations:** Department of English, College of Arts, King Faisal University, Al Ahsa, Saudi Arabia; Sichuan Agricultural University, CHINA

## Abstract

This paper investigates the online discourses on “sustainability” and explores how environmental sustainability has been constructed within the Saudi online discourse. In doing so, the study focuses on the potential of the Saudi Green Initiative (SGI), along with Green Riyadh and King Salman Park, to promote sustainability awareness in the kingdom. The corpus of the study comprises news articles and Twitter hashtags. In addition, the study uses corpus-based discourse analysis to examine the keyness and intertextuality. The results of the study convey a contextualized national identity while communicating internationally within discursive circles. It is also identified that interdiscursivity is central in the corpus, although the discourse of action especially dominated other smaller discourses, such as consequences/risks, economy, and tourism. The results also indicate the prominent construction of officials, which could be linked to the dynamics of change in the Saudi scene. Finally, the study emphasizes the action-oriented nature of such discourse while drawing attention to some of the challenging issues to long-term advocacy in the country.

## 1. Introduction

The media plays a significant role in shaping and constructing public discourses by reflecting on various concepts, opinions, and ideologies [1]. Castells [2], (p. 316) for instance, argues that visibility in the media is essential in transforming environmental sustainability "from a condition into a public problem and then into a policy concern." This is significant because increasing public awareness of the issue of environmental sustainability is necessary in order to modify the social behaviours and institutional frameworks that contribute to and magnify the problem. Climate change, for instance, is not the product of individual actions and preferences; rather, it is the outcome of high-carbon systems that have been historically ingrained, and which have generated and maintained powerful patterns of social life. This article looks at the most prevalent linguistic patterns in discourses within environmental sustainability in the Saudi online media.

Understanding and conceptualizing the notion of discourse might offer insight into the significance and rationale of discourse studies within the field of environmental sustainability. The term ‘discourse’ is defined as language-in-use—that is, language enacted “on-site” [3] (p. 7) to signify different activities and identities and, more broadly, to construct themes while serving different functions. Halliday explains the three meta-functions of language as [4, 5]: ideational, interpersonal, and textual. The ideational function relates to the communication of content and information, while the textual function focuses on linguistic tools, such as cohesion and coherence. Fairclough [6] (p. 134–135) takes language use as communicating “(i) social identities, (ii) social relations and (iii) systems of knowledge and belief.” However, van Dijk [7] views discourse as operating on three levels simultaneously—micro, meso, and macro [8]—which correspond respectively to the research questions presented later in this paper.

We can envision the links between discourse and society on the one hand and the epistemology of constructivism on the other, with language profoundly shaping individuals’ perception/construction of reality [9] and vice versa. Consequently, fundamental notions related to this framework—particularly those relevant to our research, such as developing sustainability or climate change adaptation—can be viewed as discursive constructs [10, 11] in that their construction is always subject to dynamic discursive practices at the three overlapping levels mentioned above. Different studies have established the social constructivist view of environmental discourses decades ago (see Hansen [12], Allen et al. [13]), and its relevance persists in work literally investigating the strong causal relationship between linguistic patterns and human survival [14]. In addition, Allen et al. [13] demonstrated this discursive construction by drawing on a 2007 speech by the CEO of Walmart that was influential in initiating the world’s largest retailer’s sustainability measures, whose efforts were further enhanced in subsequent speeches. Due to the discursive power attached to discourse, the aforementioned Walmart speech became a driving force to promote sustainable activism in the retailer’s policies. By the same token, the discourse examined at this study has similar potential in constructing context, shaping action and promoting sustainable awareness.

### 1.1. Understanding sustainability: Localization and appropriation

Sustainability began attracting attention as a concept with the Brundtland Report in the 1980s [15] (p. 8). Sustainable development is defined as meeting “the needs of the present without compromising the ability of future generations to meet their own needs.” Some viewed the emergence of this term as eclipsing the key concept of environmental discourse in the 1970s [16]. Sustainability thus replaced “crisis-driven” perceptions of the environmental movement, which focused heavily on threats with a “biding sense of urgency” (p. 59). This alternative became a “mainstream word” by 2010 (p. 58) and was ultimately recognized as a “cultural” or “sociopolitical” buzzword [17] (p. 10). As such, we can perceive the emergence of sustainability as a counter-discourse corresponding to enlightenment-based modernity and progress [18]. On this view, sustainability can be construed as a reaction to capitalist discourses promoting overproduction and excessive material consumption for the sake of economic growth.

However, over the past three decades, sustainability has been criticized for its “vagueness” [19] (p. 400). This appeal can be traced back to its interdisciplinary nature. Following this logic, Leitch and Davenport [20] suggest that sometimes this ambiguity can be strategically beneficial as it allows for the coexistence of multiple interpretations of sustainability, thus promoting diversity and defying limitations. However, for this to take place efficiently, sustainability must be appropriated and recontextualized precisely [21, 22].

Globally, it is essential to promote the localization of sustainability. This has inspired the current research examining the meaning found in negotiations taking place within Saudi discursive practices. Promoting public awareness of sustainability’s diverse components has always been necessary to localize such efforts efficiently, which is especially critical in developing countries [1, 22]. Castells [2] (p. 316) highlights how discursive practices (e.g., news discourse) transform sustainability from a “condition to a public issue to a policy concern.” Recently, Saudi policymakers have intensively addressed sustainability. This mirrors the objectives of Saudi Vision 2030 [23], issued on April 25, 2016, as a transformative agenda emerging from a sustainable Saudi vision. While this vision encompasses the different components of sustainability, this study investigates only the environmental aspect—namely, the Saudi Green Initiative (SGI)—along with two other governmental projects operating along the same lines—the Green Riyadh (GR) and King Salman Park (KSP). The SGI was officially launched on October 24, 2021, to improve quality of life in Saudi Arabia, and protect future generations by achieving net-zero carbon emissions by 2060 [24].

Against this backdrop, this article evaluates the digital rhetoric on "sustainability" and uncovers how environmental sustainability has been formed within the Saudi online discussions while paying attention to the importance of intertextuality and keyness. The focus of the study lies on the environmental aspects to explore the potential of the Saudi Green Initiative (SGI), as well as Green Riyadh and King Salman Park, to promote sustainability awareness throughout the kingdom.

### 1.2. Previous studies

Many researchers have conducted corpus-based studies to investigate print media discourses using theoretical insights of critical discourse analysis, discourse analysis and corpus linguistics [25, 26]. This study is novel in integrating the theoretical underpinnings of ecolinguistics and corpus linguistics to investigate the environmental component of sustainability discourse in Saudi Arabia. Ecolinguistics is defined as “the study of the impact of language on the life-sustaining relationships among humans, other organisms, and the physical environment” [27] (p. 105). Therefore, this study undertakes the ecological discourse analysis suitable for analyzing the discourses of the environment. Stibbe [28] places ecological discourses on a continuum from destructive to ambivalent to beneficial. Destructive discourses emphasize excessive natural exploitation and are often proffered as a form of resistance to such destruction, as examined in [29]. On the other hand, the stand of ambivalent discourses is mixed, whereby they may be ecologically motivated but still fail to maintain politically correct discursive practices, as examined in [30, 31]. Most relevant literature covers these two categories. Therefore, this study is significant as it highlights the very important issue pertaining to the discourses of environment embedded with eco-friendly ideologies and about environmental issues. These discourses can be viewed as linguistic strategies or “alternative ways” of constructing the world [32] (p. 391). This categorization scheme can also be linked to a distinction often made in discourse studies between critical analyses resisting the first two categories and positive evaluations of the third [8].

Ecological discourse analysis—whether critical, positive, or both—has transformative potential; meanwhile, discourse as an interdisciplinary concept with a multimodal semiotic nature can take several forms, among which media are the most prominent. Indeed, much of the relevant literature is based on traditional forms of media (e.g., newspapers) [26] or new forms of media (e.g., Twitter) [33] as manifestations of sustainable activism. For instance, Dayrell [34] and Nambiar [22] offer ecological analyses of Brazilian and Indian discourses, respectively, in which the power of media helped promote environmental awareness. However, at the time of writing, studies on the sustainability discourse in the Saudi context were virtually nonexistent. Despite tremendous interest in sustainability-related issues in various disciplines, discourse studies (environmentally oriented or otherwise) are difficult to find.

Within the discourse analysis literature, a plethora of empirical frameworks have been proposed, corresponding to the diversity of motivations and rationales in the relevant fields. These include interdisciplinary discourse works such as [35] as well as corpus-assisted discourse studies (CADS) [36], analyzing both quantitative and qualitative data. This is a combination of corpus linguistics (wherein large linguistic texts are stored automatically and analyzed electronically by specifically designed software searching for linguistic patterns [37]) and discourse analysis (a critical approach offering interpretations within larger contexts). Corpus tools have been used extensively in discursive studies in the last decade (e.g., [38, 39]), and many ecolinguistic studies have been inspired by CADS [26, 29, 34, 39–41]. This study fell within the scope of CADS and addressed the Saudi online environmental sustainability discourse’s construction as well as the dynamics operating within its discursive circle.

Increasing public awareness regarding environmental sustainability is currently a priority in Saudi Arabia [23]. Consequently, many policy reforms and transformative initiatives have taken place during the last decade in accordance with the Saudi Kingdom’s aspirations. Thus, the current study linguistically examined how the Saudi government is constructing its environmental sustainability discourse through online platforms, addressing three research questions:

- RQ1—How is environmental sustainability constructed in Saudi official online discourse in terms of keyness?
- RQ2—How is environmental sustainability constructed in Saudi official online discourse in terms of intertextuality?
- RQ3—What are the implications of such constructions?

This paper is divided into five sections: introduction, material and methods, results, discussion, and conclusion. Section 1 establishes the study’s theoretical grounding, examines the concept of sustainability, and localizes it within the Saudi scene.

## 2. Materials and methods

### 2.1. Corpus compilation

The study uses a self-built corpus, Saudi online environmental sustainability corpus (SOESC) of online texts to address the research questions. The corpus is compiled from online sources freely available to public on the official website of the SGI; and it did not violate its terms and conditions since none of these sources is reproduced here but they were rather disjoined as separate words and phrases using corpus tools. The corpus contained 87,237 words and was divided into two sub-corpora. The first sub-corpus was collected from www.saudigreeninititaive.org [24] in November 2021, which was selected to represent the Saudi online environmental discourse for several reasons. First, the SGI was launched as a comprehensive forum encompassing different topics relevant to environmental sustainability in the Saudi scene, documenting, distributing and circulating its content to popularize science. Second, the initiative is more internationally focused than other schemes in Saudi Arabia regarding scope and target, evident in the website’s multilingual interface, which communicates content in Arabic, English, Chinese, and Japanese. Some website materials, such as the “About,” “Events,” and “Targets” sections, were identical to those in the second sub-corpus. Hence, to avoid double-posting, the study focused only on the “News” section, which contained national and international news articles that the SGI reposted. All 36 articles (from 17 different sources) available in this section were extracted in November 2021, transformed into individual corpus-friendly plain-text files, and included in this first sub-corpus (SOESC1 hereafter) comprising 33,712 words. Table 1 illustrates the details of the SOESC; “type” refers to the word entries in a single unit, while “tokens” refers to the actual number of occurrences of these word entries [25].

**Table 1:** The SOESC in detail.

SOESC	Tokens	Types
SOESC1	33,712	5,051
SOESC2	53,525	4,964
Total	87,237	8,169

The second sub-corpus (SOESC2) was extracted in December 2021 from the SGI’s official Twitter account (@Gi_Saudi). This account had amassed roughly 107,000 followers since it was launched in March 2021. The account tweets in both English and Arabic with identical content, meaning that Arabic tweets are often followed by English translation tweets and vice versa. Therefore, to avoid double-posting in the corpus, and to exclude the translation variable, only one language—English—was included. This decision was also intended to maintain consistency of content between SOESC1 and SOESC2. Accordingly, all the English tweets were extracted from the account. A preliminary data compilation comprises King Salman Park (@Riyadhksp) and Green Riyadh (@greenriyadh_sa). Like the official RGI Twitter account, both of these accounts were officially launched by the Saudi government in February 2019 and are bilingual in their content. As such, they were included in SOESC2 (only the Twitter accounts and not their corresponding official websites), mainly because of identical posts on official websites and their corresponding Twitter accounts.

Additionally, as hashtags are used repeatedly by the accounts and an inherent feature of online communication and microblogging on social media platforms, especially Twitter, SOESC2 included several hashtags: #saudigreeninitiative, #SGIforum, #Kingsalamanpark, and #greenriyadh. All Twitter data were extracted from Twitter Premium API using the third-party tool TrackMyHashtag. These data were later cleaned and transformed into plain-text files prior to uploading to the corpus software. By combining the two sub-corpora, the SOESC incorporated posts created by local Saudi institutions and international posts from other actors whose content aligned with that of these national governmental institutions.

### 2.2. Wmatrix5: A web-based corpus processing environment

This study adopted an inductive methodology to examine the keywords comprising the SOESC. This contrasts with deductive analysis, which begins with a priori assumptions of what lexical items constitute a given discourse. Drawing on the principles of grounded theory, this study’s corpus-based methodology identified the starting point of analysis for this dataset. Specifically, the corpus-processing software Wmatrix5 searched for linguistic patterns [42]. While some studies primarily utilize corpus tools operating on simple frequency lists [39, 43], in the current dataset, this was expanded to keyword analysis. CADS compare a given corpus with a reference corpus to highlight not only the most frequently occurring words but also words that are especially relevant to the comparison factor [25]. Keyness, as opposed to frequency, can give more insight into what linguistic patterns distinguish a given corpus. By carrying out a keyword analysis, this study should be able to identify discursively what features the Saudi online discourse on environmental sustainability has and what implications this leads to.

Further, this study uses British English 2006 (BE06) as the general reference corpus to generate keyword lists, mainly because of the availability of BE06 within the Wmatrix5 interface as well as its inherent genre- and style-related features. These keywords’ statistical significance was calculated using the log-likelihood (LL) test to ensure that their keyness was not due to chance [25]. Such keywords were then categorized semantically and analyzed separately based on their grammatical parts of speech. At this stage, insights derived from some linguistic analytical tools, such as the appraisal system [44] and process type analysis [5], were utilized. After the generation and classification of these keywords, some other tools in Wmatrix5, such as concordance lines and semantic key concepts, were employed. Concordance lines examine a given keyword’s linguistic context. Semantic key concepts were used to determine the semantic domains assigned to the corpus’s content. This automatic process assessed the study’s reliability, as it allowed for comparison of the software’s categorization with that performed by the author [45].

The second phase of the analysis targeted another linguistic indicator—intertextuality—as a discursive tool. Intertextuality analysis is concerned with how “texts are linked to other texts” [46] (p. 84). Online texts, such as those comprising the SOESC, are inherently intertextual. Zappavigna [47, 48] emphasizes that fundamental features, such as the use of hashtags (#) and mentions (@), often create discursive bonds and affiliations within discourses. This analysis phase utilized these features while building upon findings from the first phase. This inductive approach allowed for the examination of cumulative evidence as it gradually deconstructed the Saudi online discourse on environmental sustainability.

## 3. Results

### 3.1. Keyness: Keywords

The keyword analysis using Wmatrix revealed thousands of words of statistically significant relevance in the SOESC, many more than in BE06. However, note that corpus studies typically focus on content words like nouns and verbs as they are more indicative of a text’s “aboutness” [25]. In contrast, grammatical words such as prepositions, articles, and pronouns, are often indicative of style and not content [49]. Accordingly, all grammatical words were excluded from the keyword list because of the study’s focus on content; nevertheless, this list was still too extensive for a manual discourse analysis. Consequently, the top 100 key content words were further analyzed and later classified into different parts of speech (nouns, verbs, adjectives, or adverbs) for clarity of presentation as well as investigating any meaningful patterns of their distribution. Table 2 shows these organized in descending order of keyness, calculated using the LL test. Before exploring these results, note that the top five keywords were general indicators of online discourse and microblogging. Based on the presence of *RT* (i.e., retweet), *HTTPS* (hypertext transfer protocol secure), two hashtags, and a mention, the heavily digital nature of the SOESC was evident.

**Table 2:** Top 100 keywords in the SOESC in descending order of keyness.

No.	Word	No.	Word	No.	Word	No.	Word
1	*RT*	26	*2030*	51	*Joined*	76	*100 million*
2	*https*	27	*Efforts*	52	*Significantly*	77	*Conservation*
3	*#saudigreeninitiative*	28	*Environmental*	53	*crown prince*	78	*Coastline*
4	*@gi_saudi*	29	*Change*	54	*@rbalsaud*	79	*Mangrove*
5	*#sgiforum*	30	*Achieving*	55	*Countries*	80	*Help*
6	*Saudi Arabia*	31	*Land*	56	*Initiatives*	81	*#climateaction*
7	*Climate*	32	*Bin*	57	*Forum*	82	*Sea*
8	*Global*	33	*Trees*	58	*Arabia’s*	83	*Ambitious*
9	*HRH*	34	*Targets*	59	*Minister*	84	*lemon trees*
10	*2030*	35	*Challenges*	60	*Riyadh*	85	*#cop26*
11	*Green*	36	*Addressing*	61	*Ecosystems*	86	*#saudi*
12	*Initiative*	37	*#crownprince*	62	*CEO*	87	*achieve*
13	*Action*	38	*Confronting*	63	*#climatechange*	88	*Crown Prince Mohammed*
14	*Emissions*	39	*30by30*	64	*New*	89	*offset*
15	*global ocean*	40	*@abdulazizsna*	65	*Methane*	90	*targeted*
16	*Announced*	41	*Recap*	66	*Tons*	91	*#mgisummit*
17	*Kingdom*	42	*Pillar*	67	*Sustainable*	92	*today*
18	*30%*	43	*Alliance*	68	*@arabnews*	93	*Reema Bandar al Saud*
19	*Saudi*	44	*Represent*	69	*Plant*	94	*tourism*
20	*Kingdoms*	45	*Working*	70	*Mangroves*	95	*increase*
21	*Carbon*	46	*2060*	71	*Begins*	96	*#greenriyadh*
22	*Protect*	47	*Planted*	72	*KSA*	97	*representing*
23	*Energy*	48	*#saudiarabia*	73	*Stabilize*	98	*planet*
24	*Contribute*	49	*Salman*	74	*96 million*	99	*future*
25	*Vision*	50	*60*	75	*Forests*	100	*opportunity*

#### 3.1.1. Nouns

**Fig 1:**
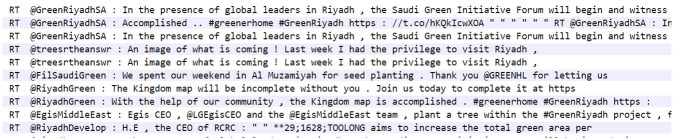
Concordance lines of *RT*.

Table 2 shows the top 100 keywords in the SOESC in descending order of keyness revealing the significant themes constructed in the SOESC. Six key themes (three major, three minor) were expressed with varying degrees of prevalence. The first major theme was “action,” demonstrating that the SOESC had an activist aspect, with the frequent occurrence of words such as *#saudigreeninitiative*, *#mgisummit*, *action*, *global ocean* (part of the term referring to the “30 by 30” global initiative), *#cop26*, *efforts*, *change*, *alliance*, *vision*, *conservation*, and #*climateaction*. In fact, the most prevalent keyword on this list, *RT*, is inherently related to activism, as this built-in feature of Twitter allows users to further publicize tweets. While retweeting does not necessarily entail support for the content of that tweet, examination of a sample of 1,000 concordance lines for its 28,428 occurrences clearly demonstrated this activist function. Fig 1 shows some concordance lines of RT.

The second major theme in the SOESC was “natural landscape.” Naturally, words related to greening and the natural environment, such as *ecosystems*, *trees*, *plants*, *mangrove*, *mangroves*, *forests*, and *lemon trees*, were heavily represented. Furthermore, included under this theme were words related to the aquatic landscape, such as *sea* and *coastline*. *Climate*, as a standalone noun, was also included in this category, as it appeared in contexts different from those of other keywords, such as #*climateaction* and #*climatechange*. Interestingly, however, the third major theme, “officials and decision makers,” appeared often in the SOESC. While it is common for proper names, titles, and positions of authorities to appear within such discourse, assigning such prominence to this theme could be indicative of transforming dynamics in Saudi Arabia. *HRH* (i.e., “his royal highness”) was most prevalent within this theme, along with *#crownprince*, *#crown_prince*, and *#crown_prince_Mohammed*. Furthermore, proper names and official accounts were also highly prevalent, such as *bin* (Arabic translation for “son of,” often used in proper names), @*abdulazizsna* (Minister of Interior), @*rbalsaud* (Saudi ambassador to US) and *Reema Bandar Al Saud* (same as @rbalsaud). Functionalizations connecting individuals with their roles (i.e., official positions), such as minister and CEO, were also common.

**Fig 2:**
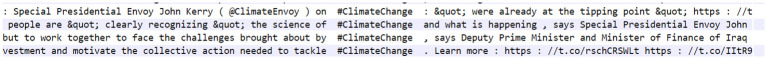
Concordance lines of #climatechange.

**Fig 3:**
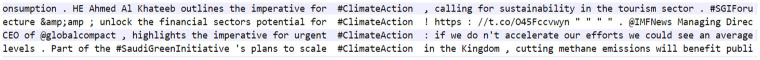
Concordance lines of #climateaction.

At the periphery of these major themes lay some minor overlapping themes. Intriguingly, the theme of “consequences” was less commonly represented in the SOESC than the theme of “action” required in response to such consequences. Keywords such as *emissions*, *carbon*, and *methane* were part of this theme, and examining their concordance lines revealed their negative semantic associations. It also revealed high collocations, which occur when words preferentially exist near each other. Furthermore, *#climatechange* was included in this category, with clear strong associations with the negative consequences of human actions. Figs 2 and 3 demonstrate the uses of *#climatechange* and *#climateaction*.

The second minor theme was “nation,” referring primarily to Saudi Arabia. Keywords such as *Saudi Arabia*, *Kingdom*, *Arabia*, *Riyadh*, and *KSA* comprised the Saudi identity within this discourse. A relevant keyword, *countries*, also appeared on this list. Nevertheless, the term’s rather general meaning did not refute the specific focus on Saudi identity. Finally, the third minor theme was “numbers.” The SOESC contained different numerical expressions; first, there was *2030*, the concordance lines of which revealed strong connections with the Saudi Vision 2030 and, to a much lesser extent, the global 30 by 30 initiatives. The terms *30%* and *30 by 30* also appeared frequently, which explicitly referred to the aforementioned initiative. Similarly, *2060* and *60* were used heavily as references to another initiative aiming for net-zero emissions by 2060. The concordance lines of *96 million* and *100 million* revealed strong associations with retweets quantifying the tons of carbon offset and the number of mangroves to be planted within the SGI, respectively. Hence, the numbers’ theme overlapped heavily with the action theme; the exception was *96 million*, which overlapped with the consequences theme. The occurrences of such keywords shows the significance of environmental issues and the government is paying much attention to provide a sustainable environment.

#### 3.1.2. Verbs

While verbs occur relatively less frequently in keyword lists compared to nouns, examining them using corpus tools can still reveal important patterns. But in this study, rather than classifying verbs according to their semantic meanings, the researcher used Halliday’s [4, 5] concept of the transitivity theory. This allowed the researcher to categorise verbs in a way that better reflects their functions. The world is understood to be composed of both internal and external experiences when viewed through the lens of this paradigm. Each procedure, as exemplified by the verbs, involves participants with varying capacities to bring about change within either experience, or to prevent such change from occurring. Within this framework, we came up with six different possible process types. The first verb on the keyword list was *announced*, the concordance lines of which indicated that it was a verbal process of saying. Another verb, with moderate keyness, was *represent*. However, unlike *announced*, which signifies a more tangible change in the external experience, *represent* is more of a relational verb. Relational processes involve no action on the participant’s part, as they denote a state of being or characteristic of one’s identity (as demonstrated in the SOESC). Apart from these two verbs, most others were explicitly material processes, signifying the highest level of action. Such processes primarily result in a (purportedly) tangible change in the external experience. To illustrate, *protect*, *contribute*, *change* (as a verb), *achieving*, *working*, *planted*, *joined*, and *begins* all are examples of such action. This was consistent with the prominence of the action theme established via nouns and demonstrated an action-based agenda within the SOESC. This was further supported by the fact that the keyword list did not reveal any examples of mental processes, which are less noticeable, more internal activities.

#### 3.1.3. Adjectives/Adverbs

Because these two aspects of speech emerged much less frequently in the SOESC, they were combined into one category. This was also because both adjectives and adverbs tend to describe what is often already established by co-occurring nouns and verbs. According to Huddleston and Pullum [50] (p. 20),‘adjectives inherently describe properties or states related to people or things (abstract or concrete)’, and the most obvious forms of adverbs are those derived from adjectives by adding the suffix -*ly*. Clearly, not all adverbs are derived this way; nevertheless, this should signify the strong bonds between these two classes. The only difference is on the functional level in which adjectives are modifiers of nouns (or noun phrases), while adverbs often modify verbs (or verb phrases), adjectives, or other adverbs.

Seven adjectives and two adverbs appeared in the top 100 keyword list, and despite their limited occurrence, they were analyzed using the approach presented earlier. However, interestingly, when attempting to relate these to the appraisal system [44] of evaluative language, five of the nine keywords in this category did not reveal any evaluative content. Within the appraisal system’s framework, evaluation instances are often sought to decode semantic systems on an interpersonal level. As adjectives and adverbs are inherently modifiers, they lend themselves to investigations within such a framework. However, modifiers such as *global* and *Saudi* were more informative of types than they were evaluative. *Green* and *environmental* were similar in this regard and appeared in many concordance lines consistent with the themes of action and natural landscape. *Today* is an adverb related to time and was used heavily in the SOESC without setting any evaluative tone. Examining its concordance lines illustrated that this adverb occurred most frequently in the context of the action theme.

**Fig 4:**
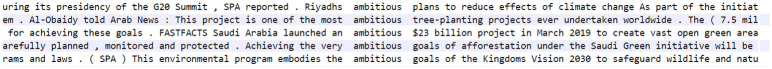
Concordance lines of *ambitious*.

*Sustainable* was another predominant adjective on the keyword list; however, unlike *environmental*, which could be considered a close synonym, *sustainable* demonstrated a degree of evaluation, along with its informative content. In terms of attitude, the semantic system is concerned with feelings as a system of meaning. For the judgment component, the evaluation is concerned with ethics (i.e., attitudes expressed toward a particular behavior). Inevitably, such expressions can have either positive or negative valence. Analyzing *sustainable*’s use in the SOESC revealed a more positive orientation as it coincided primarily with the action theme. *New* and *ambitious* also demonstrated the judgment component of the action theme, but with more explicit positive valence due to their semantic implications. Fig 4 shows the concordance lines for *ambitious*, which suggest the action theme’s positive construction (relating to hope and aspirations).

**Fig 5:**
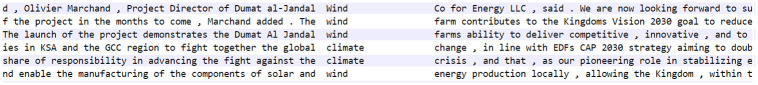
Visualization of SOESC keywords generated by Wmatrix5.

It is also noteworthy that this list identified most of the function words as underused. These were intentionally excluded from the analysis, but this finding could have additional implications for the SOESC. The keyness analysis identified words used far less in the corpus under examination (SOESC) than in the reference corpus (BE06). While this is a stylistic feature associated with the SOESC’s news/microblogging genres, it also indicates its informative nature. Fig 5 is a visualization of the top 100 keywords appearing in the SOESC, including function words. The larger the word, the more statistically significant the keyness it exhibited. Italics denote underused words.

### 3.2. Keyness: Key concepts

The previous section examined which individual words were key using corpus tools and linguistic analyses. What follows is a key semantic concept analysis, a method of automatic processing performed via Wmatrix5. Table 3 shows 88 semantic terms identified via this process. Examination of these key concepts along with their concordances also identified the semantic themes that were identified manually, supporting the latter method’s reliability. Notably, some semantic key concepts s (e.g., *weather*) occurred in different themes instead of being confined to one theme only.

**Table 3:** Major and minor themes identified and corresponding key semantic concepts identified using Wmatrix5.

	Theme	Key Semantic Concepts
Major	Action	Time: future, weather, farming and horticulture, helping, general action/making, wanted, time: new and young, giving, money generally, avoiding, color and color patterns, trying hard, change, linguistic actions: states and processes, communication, participating, health and disease, time: beginning, no change, substances and materials: solid, learning, difficult, work and employment: generally, science and technology in general, life and living things, business: generally, mental object: conceptual object, industry, reciprocal, important, time: beginning, hindering, cause and effect/connection, alive, evaluation: good, inclusion, temperature, important, non-governmental, expect, evaluation, healthy, time period: long, children’s games and toys, cheap, chance, luck
Major	Natural landscape	Geographical terms, green issues, weather, farming and horticulture, plants, the universe, color and color patterns, places, sailings, swimming, life and living things, darkness, alive, long, tall and wide, residence, temperature, living creatures: animals, birds, healthy
Major	Officials/Decision makers	Government, other proper names, respected, personal names, in power
Minor	Consequences	Weather, substances and materials, change, health and disease, no change, cheap, substances and materials: solid, difficult, science and technology in general, substances and materials: liquid, no respect, industry, temperature: hot, on fire, hindering, cause and effect/connection, temperature, non-governmental, expect, evaluation, time period: long
Minor	Nation	Geographical names, places
Minor	Numbers/Quantities	Numbers, measurement: weight, size: big, quantities: little, measurement: area, measurement: speed, measurement: size

**Fig 6:**
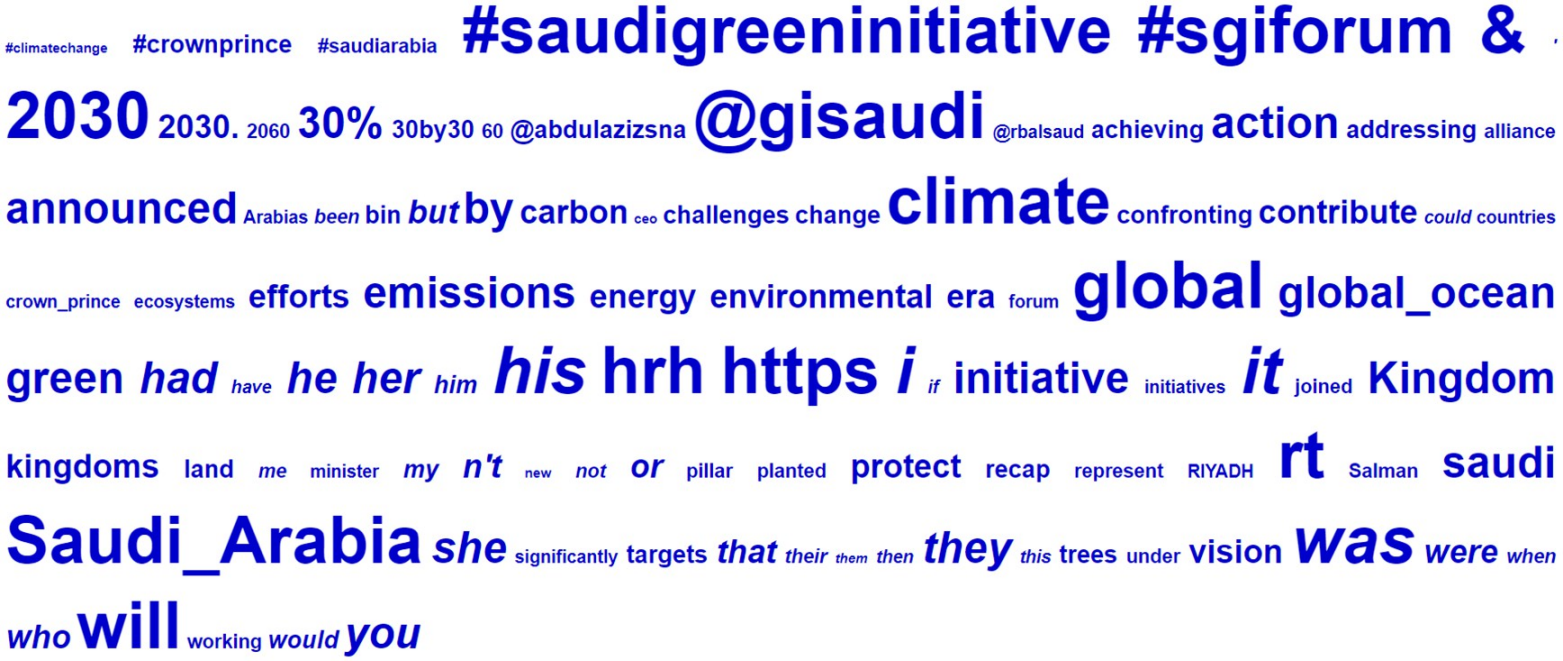
Concordance lines of weather as a key semantic concept.

Such concepts occurred within the action theme in discussions calling for climate action, within the consequences theme when highlighting the risks associated with climate change, or even when simply referring to weather manifestations (e.g., wind, dust, sand). Fig 6 displays several concordance lines belonging to one semantic key concept (*weather*) while spanning different themes. Another issue with automatic semantic annotation was categorizing words with metaphorical meanings such as “players” which was included within climate action under the semantic tag of children’s games and toys. Further, Wmatrix5 could not semantically match several keywords, including acronyms (e.g., KSA [Kingdom of Saudi Arabia]), mentioned usernames starting with @, or hashtags starting with #. Finally, certain words (e.g., room, rooftop, corridors) were used both literally and metaphorically, and as with the “players” above, they were assigned automatically to the semantic key concept of *parts of a building*. However, their concordances were so diverse that assigning them to one or two themes was not feasible.

These findings should not discourage the use of automatic semantic annotation tools but instead remind us that they should be used in conjunction with other manual analysis tools. In fact, if not for the automatic semantic tagger, exhaustive analysis of the entire SOESC would have been impossible. To illustrate, a seventh theme could be identified that did not appear significant when examining the top 100 words: *economy*. Ranking 15th on the list of 88 key semantic concepts, the theme of *economy* was highly salient in this corpus. It was strongly associated with the action theme, as many of its concordances related to spending on protective or corrective measures by national and international organizations or banks.

### 3.3. Intertextuality

#### 3.3.1. Intertextuality: Hyperlinks in SOESC1

As explained previously, intertextuality is a significant linguistic tool with major implications for the discursive level. The very act of incorporating several news articles from different sources is inherently intertextual [51]. Table 4 shows the included sources.

**Table 4:** National and international sources appearing in SOESC1 (frequency).

National	International
Alarabiya News (3)	Gulf News (1)
Arab News (9)	The National (3)
	Bloomberg (2)
	Climate Change News (1)
	Deutsche Welle (1)
	Forbes (1)
	France24 (1)
	Mashable MG (1)
	Reuters (8)
	S&P Global (1)
	Sputnik News (1)
	The Guardian (1)
	The Independent (6)
	Russia Today (1)

It is evident that the SOESC1’s international nature was constructed at a discursive level. For example, while the Saudi identity was established at the keyness level, it was communicated both internationally and locally. More than 70% of reposted English news items originated from non-Saudi sources. Thus, we can assume that such content was tailored for an international audience. This explains why English was the primary language of communication besides Arabic (the country’s official language). Additionally, it is noteworthy that while this list featured general news sources, such as *Reuters* and *The Guardian*, other sources with a more specialized focus were also present. Some have economics-related agendas, such as Bloomberg, S&P Global, and Forbes, while others are more concerned with environmental sustainability, such as *Climate Change News* and *World Energy Council*.

#### 3.3.2. Intertextuality: Mentioned usernames in SOESC2

Intricately interwoven within intertextuality is the inclusion of other individuals/institutions within the same discourse. While this feature impacts online discourse heavily, only its most explicit manifestation—mentioned usernames—is examined here. As a built-in feature of microblogging, mentioned usernames (often referred to as “mentions”) can be quite informative about how discourse is distributed among diverse actors. Table 5 shows the most frequently used usernames in the corpus as identified by Wmatrix5. It is also important that while these usernames included those mentioned by individuals working officially with the SGI, GR, or KSP, the top 10 usernames in the Wmatrix5 keyword list also included those mentioned by anyone posting with the hashtags. However, Neom (a planned Saudi “smart” city and prospective tourist attraction) and the Red Sea Development Company (another Saudi organization responsible for regenerative tourism) exemplified an eighth theme within the SOESC: *tourism*. The official accounts of the U.S. Mission to KSA and the American Chamber of Commerce in Saudi Arabia illustrate another interesting link. While Saudi identity was established primarily via keyness analysis, intertextuality analysis revealed that this was communicated internationally, as illustrated by the mentioned American usernames. This occurred primarily in discussions of an event in which the U.S. Mission planted trees as part of the GR project.

**Table 5:** Top mentioned usernames in the SOESC.

Account/Corpus	Top Mentioned Usernames
@gi_saudi	@gi_saudi @neom @moenergy_saudi @theredseaglobal @kaust_news @mewa_ksa @riyadhdevelop
@greenriyadh_sa	@usainksa @amchamksa @riyadhdevelop
@riyadhksa	@riyadhdevelop
Wmatrix5 keywords	@gi_saudi @abdulazizsna @rbalsaud @arabnews @independent @rcu_sa @mewaksa @neom @unfccc @moenergysaudi

In addition, the results showed a similar pattern, including Saudi official environmental institutions’ predominance. This analysis extended to the international level by including UN Climate Change’s official account. The theme of *tourism* emerged via the keyword *@rcu_sa*, the official account of the Royal Commission for AlUla, the world’s largest cultural oasis and a major tourist attraction in Saudi Arabia. In keeping with the *decision makers/officials* theme, the Saudi Minister of the interior and the Saudi Ambassador to the U.S. were featured here. Finally, two news outlets—the Saudi *Arab News* and the international *Independent*—were keywords, mirroring the links established within SOESC1.

#### 3.3.3. Intertextuality: Hashtags in SOESC2

Hashtags were present in the top 100 keywords list (Table 2); however, because some were already examined in the keyness analysis, the top 20 hashtags were also included. This broadened the analysis of intertextuality within SOESC2; while some hashtags might not have been significant in terms of keyness, they could potentially offer insights into practices within the discourse. Most of the hashtags included in Table 6 correspond to the major and minor themes identified in Sections 3.1 and 3.2, but the hashtags further down the list also demonstrated the tweeted content to be inherently intextualized and intextualizing regarding the themes of *economy* and *tourism*. The former was exemplified by *#oott* (Organization of Oil Trading Tweeters) and the latter by *#alula*. Interestingly, the content’s news-like nature was made apparent when including *#spagov*, which was intextualized with the Official Saudi Press Agency. Further, including *#mgisummit* and *#mgi*, which refer to the Middle East Green Initiative, geographically broadened the tweeted content. The MGI is another initiative being launched by Saudi Arabia alongside the Youth Green Initiative (whose hashtag appeared further down the list) and the SGI for environmental advocacy and literacy. This created the same pattern of Saudi identity already established, but this time it was intended to be circulated internationally.

**Table 6:** Top 20 hashtags in SOESC2.

Hashtag	Occurrences	Hashtag	Occurrences
*#saudigreeninititive*	22026	*#biodiversity*	828
*#sgiform*	15220	*#riyadh*	811
*#crownprince*	1816	*#netzero*	772
*#saudiarabia*	1658	*#vision2030*	625
*#climatechange*	1282	*#kingsalmanpark*	546
*#climateaction*	1019	*#sgforum*	411
*#cop26*	995	*#oott*	397
*#saudi*	987	*#spagov*	364
*#mgisummit*	958	*#alula*	352
*#greenriyadh*	904	*#mgi*	296

The findings mentioned in each section were correlated, as they revealed recurrent patterns at different levels of discourse; however, there were also some important variations. This occurred because varying levels of discourse can reveal different layers of construction. This highlights the importance of the analytical triangulation adopted here.

## 4. Discussion

The previous section outlined a thorough data analysis within the parameters set in Section 2. Based on this, the following discussion attempts to answer the research questions and contextualize the results. Regarding the first two research questions, we can argue that the SOESC showed the Saudi online environmental discourse to be action-based and information-dense. Examination of the eight themes identified through the keyness analysis confirmed this construction. While the consequences and risks that often arise in environmental sustainability discourse were addressed in this corpus, they still were minor relative to the major theme of *action*. This is consistent with the construction of such discourse as popularizing science and promoting sustainability awareness. As a demonstration of knowledge transmission, then, this discourse aligns with Beacco et al.’s [52] remarks on the shift from linear and somewhat “esoteric” (p. 278) circulation to newer dynamic discursive practices wherein media’s role is redefined. According to this conception, discourses aim to educate rather than passively shaping individuals’ beliefs.

This bears strong connections to sustainable activism, which the SOESC promoted; here, change was motivated by government initiatives directed towards citizens’ awareness. This pattern is similar to other Saudi transformational discourses in which the political establishment has initiated change subtly and gradually through a top-down process [8], given discourses’ inherently intricate constitutive and constituting nature. This could also be linked to the prominence of references to officials in both the keyness and intertextuality analyses. Engstrom [53] (p. 581) suggests that within different discourses, both actors and structures must be “legitimized” to be empowered. Frequent mention of officials could also indicate authorization, one of Leeuwen’s [54, 55] legitimation strategies. Several discourse studies [56, 57] have reported similar results. Further, Bednarek [56] contends that features of microblogging such as hashtags, tweeting, and retweeting, are inherently legitimizing, with the ability to create instant affiliations.

Regarding RQ3, the action-oriented call for sustainable practices has several implications. First, one common mechanism proposed in discourse studies is framing. Entam [58] defines framing as selecting certain approaches to reality perception and constructing them saliently. While this phenomenon might seem intentional from a media/political science perspective, it can occur subconsciously, especially in linguistic contexts [21]. According to the latter more inclusive conception, framing is inevitable [26] and is interwoven with ideological, social, economic, and cognitive contexts.

Liu and Li [39] found that framing served diverse functions in their keyword analysis. Similarly, Foust and Murphy [59] determined that the U.S. discourse on global warming news coverage was framed apocalyptically, with the paradoxical positioning of human agency. They assumed these frames to have either a crisis-based tone, outlining an urgent need for intervention, or a cosmic and fate-related tone, suggesting that humans should not be held accountable. The present study found no polarization in the SOESC. Within online governmental discourses, sustainability issues are constructed to communicate the need for further human intervention. This might explain the prominence of the *action* theme over other themes and the scarcity of evaluative trends within the keyword analysis. This framing educates the public and justifies corrective and precautionary measures—a form of sustainable activism that encourages further activism in a top-down fashion. Interestingly, however, such a framing also places relevant and controversial sustainability issues (e.g., climate change) within a scientifically established framework leaving little room for skepticism. Dayrell [34] reported similar findings in examining Brazilian media coverage of climate change, which has been quite successful in promoting local sustainability awareness and highlighting the importance of human activity in this regard. Consequently, it seems that one-sided construction, as found in the SOESC, more effectively fulfills its educational mission.

Another important finding is the plurality of discourses within the SOESC (i.e., its interdiscursivity). Reisigl and Wodak [46] (p. 90) state that interdiscursivity “signifies that discourses are linked to each other in various ways.” Hence, discourses are often “hybrid” and “open.” This is directly related to the diversity of themes identified in this dataset, implying the coexistence of different discourses of varying prominence. Discourse on sustainable activism, as demonstrated by the *action* theme, was central to the SOESC’s wider discourse. This central discourse was further supported by the *officials* theme, with evident links to political discourse, as well as the discourse explicitly communicating a Saudi identity above all, regardless of the corpus’s international readership. Several other studies have identified interdiscursivity. For instance, Rajandran [60] found interdiscursivity regarding Malaysian sustainability, with a central discourse surrounded by auxiliary discourses.

Finally, this study can be a step toward the localized understanding of sustainability in the Saudi context. While this dataset emphasized *environmental* sustainability, there is still a multiplicity of components within the Saudi discourse of sustainability. As such, besides the environmental aspect, it was possible to identify links with economic components and, to a lesser degree, social and cultural components. However, further work is needed to analyze these aspects, especially given that sustainability has recently achieved mainstream status within Saudi political, academic, and social circles. For instance, King Faisal University, a well-established Saudi public university, recently reconstructed its identity to emphasize environmental sustainability. *Ithra* (translated as “enrichment” in Arabic), the King Abdulaziz Center for World Culture, published a special issue of its magazine in January 2022 solely devoted to the relationship between sustainability and the arts. This also demonstrates the need for further research.

## 5. Conclusion

In conclusion, this study has demonstrated that the Saudi discourse on environmental sustainability has played a key role in both increasing public awareness of the environmental issues and encouraging engagement with the debate, particularly in relation to deforestation. Overall, this article has shown that the Saudi government has played a key role in both of these areas, promoting sustainable advocacy to the public and making necessary transformations. The study also revealed, however, that there was an insufficient discussion regarding the rise in emissions from the energy sector. The priorities of the government appeared to be publicizing action in the press coverage, more than highlighting risks; which could be justified on the grounds that sustainable activism should not be promoted in a fear-inducing fashion. However, this indicates that there should be more reflection on how to transform business and social practices in accordance with this agenda while still highlighting the risks motivating these measures in order to ease out their transition. Consequently, this could be a challenge to the transition -that the country is currently making- into a low-carbon economy.

This s study offered a detailed examination of how the Saudi discourse on environmental sustainability has been constructed online in terms of keyness and intertextuality. The analysis identified a central action-based discourse along with top-down initiation of sustainable activism. Several auxiliary discourses were identified as well, but they all supported the central discourse. Aiming to promote sustainability awareness, this discourse was firmly established throughout the different circles in which it was distributed. The study suggests that individuals who are dedicated to the achievement of the aim of a more sustainable world have a significant influence in the achievement of the objective of successfully integrating environmental sustainability into institutions.
